# Genome-wide screen of genetic determinants that govern *Escherichia coli* growth and persistence in lake water

**DOI:** 10.1093/ismejo/wrae096

**Published:** 2024-06-14

**Authors:** Nataliya Teteneva, Ananda Sanches-Medeiros, Victor Sourjik

**Affiliations:** Max Planck Institute for Terrestrial Microbiology, D-35043 Marburg, Germany; Center for Synthetic Microbiology (SYNMIKRO), D-35043 Marburg, Germany; Max Planck Institute for Terrestrial Microbiology, D-35043 Marburg, Germany; Center for Synthetic Microbiology (SYNMIKRO), D-35043 Marburg, Germany; Max Planck Institute for Terrestrial Microbiology, D-35043 Marburg, Germany; Center for Synthetic Microbiology (SYNMIKRO), D-35043 Marburg, Germany

**Keywords:** bacteria, environment, survival, cell envelope, stress response, chemotaxis

## Abstract

Although enteric bacteria normally reside within the animal intestine, the ability to persist extraintestinally is an essential part of their overall lifestyle, and it might contribute to transmission between hosts. Despite this potential importance, few genetic determinants of extraintestinal growth and survival have been identified, even for the best-studied model, *Escherichia coli.* In this work, we thus used a genome-wide library of barcoded transposon insertions to systematically identify functional clusters of genes that are crucial for *E. coli* fitness in lake water. Our results revealed that inactivation of pathways involved in maintaining outer membrane integrity, nucleotide biosynthesis, and chemotaxis negatively affected *E. coli* growth or survival in this extraintestinal environment. In contrast, inactivation of another group of genes apparently benefited *E. coli* growth or persistence in filtered lake water, resulting in higher abundance of these mutants. This group included *rpoS*, which encodes the general stress response sigma factor, as well as genes encoding several other global transcriptional regulators and RNA chaperones, along with several poorly annotated genes. Based on this co-enrichment, we identified these gene products as novel positive regulators of RpoS activity. We further observed that, despite their enhanced growth, *E. coli* mutants with inactive RpoS had reduced viability in lake water, and they were not enriched in the presence of the autochthonous microbiota. This highlights the duality of the general stress response pathway for *E. coli* growth outside the host.

## Introduction

Besides resulting from recent faecal contamination, strains of *Escherichia coli* and other enteric bacteria can survive and grow in the environment outside the host for prolonged periods of time [1], and a large fraction of the total *E. coli* biomass might reside in the external environment [2]. The ability of bacteria to persist outside the gut is determined by complex interactions between environmental and genetic factors [1, 3]. This is exemplified by polymorphisms observed in the *rpoS* gene [4], which encodes an alternative sigma factor RpoS (σ^S^) that mediates the general stress response [5]. Although functional *rpoS* was shown to be important for the viability of *E. coli* in seawater and soil [3, 6, 7], RpoS activity is highly variable among environmental *E. coli* strains, including some isolates with non-functional *rpoS*. This has been proposed to be a consequence of the survival-multiplication trade-off in different environments [4, 8, 9]. The importance of several other gene families has been suggested by their specific enrichment among environmental *E. coli* isolates in genome-wide association studies [8, 10], but these data have not been resolved at the level of individual genes.

As a complementary approach to these association studies, here we aimed to systematically characterize the genetic determinants of *E. coli* growth and survival in the extraintestinal environment, using lake water as a model, for an individual strain. For that, we constructed a library of randomly barcoded transposon insertions identifiable by high-throughput sequencing (RB-TnSeq) [11]. RB-TnSeq and similar approaches enable obtaining genome-wide data on fitness effects of individual genes, by identifying mutants that are enriched or depleted under particular conditions, such as growth on different carbon sources [11] or colonization of the intestine [12]. The *E. coli* K-12 W3110 RpoS^+^ strain was chosen for our study, as its analysis by RB-TnSeq largely benefits from a high-quality genome annotation of W3110 [13]. However, unlike the reference laboratory lineage, this strain has a functional *rpoS* gene, and it is commonly used to investigate RpoS regulation and biofilm formation [14–18].

We observed that *E. coli* growth and survival in lake water are affected by several well-defined functional clusters of genes. The majority of the identified gene mutations reduced the growth or survival of *E. coli* in lake water, both in the presence and in the absence of the autochthonous microbiota. However, a number of gene mutations, all of which appeared to reduce RpoS activity, were also enriched in the absence of the autochthonous microbiota.

## Materials and methods

### Strains

All strains used in this work were derived from the *E. coli* K-12 W3110 RpoS^+^ strain [14] (Supplementary Table S1). Gene deletions were performed by the λ-Red recombination system as described [19], using the pISJ8 plasmid carrying both the λ-Red system and the Flp recombinase [20].

### Water sampling

The samples of lake water were taken from the small lake (depth ~1.2 m according to the local authorities, surface area ~ 235 m^2^ measured with Google Earth) near Marburg, Germany (GPS coordinates: 50°49′50″N 8°46′49″E). It is a man-made urban lake that is used for rainwater collection with no known bacterial/chemical contamination. The surrounding land is forested and not used for agriculture. The sampling times, temperature of air and water, and pH were recorded (Supplementary Table S2). Water samples were either used in their original state or filter-sterilized through the Steritop filter unit (Merck Millipore, Darmstadt, Germany) with a pore size of 0.22 μm.

### Initial assessment of *E. coli* growth in lake water

Pre-cultures of *E. coli* W3110 RpoS^+^ [14] were grown to an OD_600_ ~1 in tryptone broth (TB) medium (10 g tryptone, 5 g NaCl per litre, pH 7) on a rotary shaker at 37°C. To identify *E. coli* cells during this initial assessment of growth in the presence of the autochthonous microbiota, *E. coli* was transformed with a pTrc99a plasmid [21] encoding green fluorescent protein (GFP) under the control of a promoter inducible by isopropyl ß-D-1-thiogalactopyranoside (IPTG), and pre-cultures were grown overnight in TB with 100 μg/ml ampicillin and 50 μM IPTG to induce GFP expression. These pre-cultures were then inoculated into the samples of lake water supplemented with 50 μM IPTG at a 1:50 dilution (resulting in ~10^7^ cells per millilitre as assessed with the flow cytometer) and incubated at the room temperature in 24-well plates without shaking. The total number of cells, and, where applicable, the number of GFP-positive cells per millilitre of lake water were measured daily by flow cytometry on the BD LSRFortessa SORP cell analyser (BD Biosciences, Heidelberg, Germany) using the high-throughput sampling system for 96-well plates. For cytometry, the cultures were mixed vigorously by pipetting and then diluted 10-fold in sterile phosphate-buffered saline (PBS; 140 mM NaCl, 2.7 mM KCl, 1.5 mM KH_2_PO_4_, 8.1 mM Na_2_HPO_4,_ pH 7.4). A 488-nm laser was used to identify the cells by measuring the scatter parameters and to excite GFP. Flow cytometry results were analysed using FlowJo software (BD Biosciences) version 10.

### Construction and characterization of the transposon mutant library

The transposon mutant library of *E. coli* W3110 RpoS^+^ [14] was constructed by inserting the randomly barcoded Tn5 system (described in detail in Wetmore *et al*. [11]) into the genome. This transposon delivery system was kindly gifted to us by Dr. Adam Deutschbauer (University of California, Berkeley). It consists of the set of plasmids carrying the transposase, the kanamycin resistance cassette, and the random 20-bp DNA barcodes [11]. Each barcode is flanked by the common binding sites U1 (GATGTCCACGAGGTCTCT) and U2 (CGTACGCTGCAGGTCGAC), enabling the amplification of the barcode sequence. This barcode, together with the kanamycin resistance cassette, is in turn flanked by the transposon Tn5 inverted repeats. When introduced into the cell, the resistance cassette and the barcode are randomly integrated into the genome by the transposase. We transformed this plasmid library into our strain of interest by electroporation, and after 4 h of recovery at 37°C in Luria-Bertrani (LB) medium (10 g tryptone, 5 g yeast extract, 5 g NaCl per litre, pH 7), the cells were plated in LB-agar supplemented with kanamycin (50 μg/ml). We repeated this procedure until we reached 500 000 colony forming units (CFU) of library coverage. The colonies were washed with TB + 20% glycerol. Some aliquots were kept for library characterization, and the rest were stored at −80°C in 1 ml aliquots. To create the comprehensive map of the library, the genomic DNA was extracted using the NucleoSpin Microbial DNA Kit (Macherey-Nagel, Düren, Germany) and sonicated to an average size of ~300 bp. The NEBNext Ultra II DNA Library Prep Kit and NEBNext Multiplex Oligos (New England Biolabs, Ipswich, Massachusetts) were used to prepare the high-throughput sequencing library. To enrich the barcode sequences in the library, the primer for U1 [11] was used in the final step of the protocol. The sequences and positions of the barcodes were then determined by MiniSeq (Illumina, San Diego, California) sequencing, using the custom script written in Python (version 3.9.12, with Biopython version 1.78) [22]. Briefly, the presence of both U1 and U2 sites, and the quality of the sequence between them, was checked (length of exactly 20 bp and Q > 30 for each base). The script name is *tnseq_phase1.py.* The reads containing the confirmed barcodes were then aligned to the *E. coli* K-12 W3110 reference genome (NCBI reference sequence: NC_007779.1) using *blastn* from the *ncbi-blast* toolkit [23] (script *BLASTN_command.sh*). The sequence and the position of each barcode were then combined into a single csv file using another custom-written Python script (*tnseq_phase2.py*). The mapping resulted in 430 849 unique transposon insertions in 3833 genes.

### Cultivation of transposon mutant library

Pre-cultures were inoculated from whole 1 ml glycerol stocks of the library into 50 ml TB medium supplemented with kanamycin (30 μg/ml) and grown at 37°C on a rotary shaker to an OD_600_ ~1. These pre-cultures were then diluted 1:50 in lake water (~10^7^ cells per millilitre) and incubated without antibiotics at room temperature in 24-well plates without shaking. Samples for further analysis were taken on Days 2, 4, and 8 of cultivation, with the sample of initial culture (Day 0) used as a control. The experiment was performed with three independent lake water samples, each in three technical replicates. The biomass from four wells of a 24-well plate, each containing 2.5 ml, was combined together to form one technical replicate. Genomic DNA was extracted using the NucleoSpin Microbial DNA Kit (Macherey-Nagel). Barcode sequences were amplified by polymerase chain reaction (PCR) using Q5 polymerase (New England Biolabs) supplemented with GC-enhancer and barcoded tru-seq-derived primers to U1 and U2 sequences (200 nM of each, forward primers carried an individual barcode to allow multiplexing and the reverse primer was common) [11]. The complete primer list can be found in Wetmore *et al*. [11] (Supplementary Table S2, primers Barseq_P1 to Barseq_P2_IT096). The amount of the template genomic DNA per reaction was 150–200 ng. The cycling conditions were 98°C for 4 min followed by 25 cycles of 30 s at 98°C, 30 s at 55°C, and 30 s at 72°C, followed by a final extension at 72°C for 5 min. The PCR reactions were pooled together (10 μl from each reaction), purified with 0.9X of AMPure beads (Beckman Coulter, Brea, California), and sequenced at the GeneCore facility (EMBL, Heidelberg, Germany) using the NextSeq 500 High platform (Illumina). For demultiplexing, we relied on the sequencing facility. The quality of the resulting dataset was assessed using FastQC [24] and MultiQC [25] software (Supplementary Table S3).

### RB-TnSeq data analysis

Barcodes were extracted from the fastq files and counted using the custom Python script (*barcode_counting.py*). Knowing the position of the barcode in a read, we extracted the barcode sequences from each read and checked whether these sequences were present in the annotation. Mismatches were not allowed, and the unmapped barcodes were discarded from the analysis. On average, 64.28% of the detected barcodes were successfully found in the annotation. The abundance of each barcode in each sample was then counted. Gene fitness was then calculated in R version 4.2.2 as described in Wetmore *et al*. [11] (script *TnSeqW3110.R*). Briefly, we first calculated the barcode fitness, defined as the log_2_ ratio of barcode counts between the treatment sample (after the incubation in the lake water) and the control sample (the initial culture). The values were normalized to the total number of all the barcodes found in the sample. Gene fitness was then calculated as a weighted average of the fitnesses of the barcodes belonging to the gene, with the weight being inversely proportional to the Poisson noise-based strain variance. The gene fitness values were then normalized for the chromosome to avoid the potential bias introduced during DNA extraction (i.e. having more molecules of DNA representing the *ori* region compared to the *ter* region). For this, the running median of the gene fitness values (window size 251) across the chromosome and the mode of the gene fitness values were subtracted from the gene fitness values. This normalization removes local biases and ensures that the gene fitness distribution is centred around zero. Corresponding formulas and detailed explanations can be found in Wetmore *et al*. [11]. Gene fitness values were then averaged across the technical replicates. Enrichment analysis was performed using the STRING database version 12.0 [26]. The same database was also used for network drawing.

### Measurement of *gadB* promoter activity

Expression of GFP under the control of the *gadB* promoter was assayed using the reporter construct from the *E. coli* promoter collection [27] in 24-well plates using a plate reader. Briefly, *E. coli* cultures were grown overnight in TB supplemented with kanamycin (50 μg/ml) on a rotary shaker at 37°C. The cultures were diluted in fresh medium to an OD_600_ of 0.02 and added to the well plates in triplicates (1 ml per well). Measurements of OD_600_ and GFP fluorescence (excitation 485 nm, emission 520 nm) were performed on an Infinite M1000 Pro plate reader (Tecan, Männedorf, Switzerland) at 37°C, every 10 min, with alternating shaking modes: 150 s linear followed by 150 s orbital, repeated twice. All measurements were corrected for a no-GFP control and a no-growth control. The activity of the *gadB* promoter is then expressed in arbitrary units (AU) as the value of the GFP fluorescence divided by the 1000 × OD_600_ value.

### SYTOX Orange live-dead staining

For staining with the SYTOX Orange dye (ThermoFisher, Waltham, MA), *E. coli* cells were grown in filtered lake water for 8 days as described above, then harvested and washed once with PBS. The dye was added to the cell suspension at a final concentration of 0.2 μM, and the mixture was incubated at 37°C for 15 min in the dark with vigorous shaking. After staining, the cells were washed three times with PBS. The percentage of the SYTOX Orange positive cells was quantified using the BD LSRFortessa SORP cell analyser (BD Biosciences) with the high-throughput system for 96-well plates in three biological triplicates (each in a technical triplicate), with a minimum of 30 000 cells measured per technical replicate. Scatter parameters for cell identification were measured using a 488-nm laser, and SYTOX Orange was excited using a 561-nm laser. To analyse the flow cytometry results, FlowJo software (BD Biosciences) version 10 was used. To assess the significance of the differences between the groups, two-sample Welch’s *t*-test was used.

### CFU quantification

Samples containing bacteria were serially diluted 10-fold in PBS and then 100 μl of three dilutions were plated on LB agar in quadruplicate using glass beads. The dilutions (typically third to fifth) were selected based on flow cytometry results. After the overnight incubation at 37°C, the resulting colonies were counted using ImageJ software [28]. Plates containing <100 or more than 500 colonies were considered unreliable. The number of CFU per millilitre was determined using the formula $CFU=\frac{N\ast{10}^d}{0.1}$ where *N* is the number of colonies per plate and *d* is the dilution.

## Results

### Experimental setup to test the growth and survival of *E. coli* in lake water

The randomly barcoded transposon mutant library of the *E. coli* W3110 RpoS^+^ strain was constructed as previously described [11] (see *Materials and methods* for details). Subsequent analysis of this library using the W3110 genome as a reference showed that it contained one or multiple insertions in 3833 genes, thus covering the vast majority of the *E. coli* K-12 genome. For the sake of simplicity, only insertions in the coding gene regions, which were assumed to inactivate the respective gene function, were considered for the assessment of the gene fitness effects.

To account for potential effects of seasonal variability, water samples were collected from the same lake at different time points (Supplementary Table S2). Samples were either filter-sterilized or left non-filtered to assess the impact of the autochthonous microbiota. To mimic the release of *E. coli* from the nutrient-rich intestinal environment into the lake water, the bacterial cultures were first pre-grown in the TB medium at 37°C, and then diluted to ~10^7^ cells per millilitre in filter-sterilized or non-filtered water samples. The cultures were subsequently incubated at room temperature without shaking for up to 8 days.

**Figure 1:**
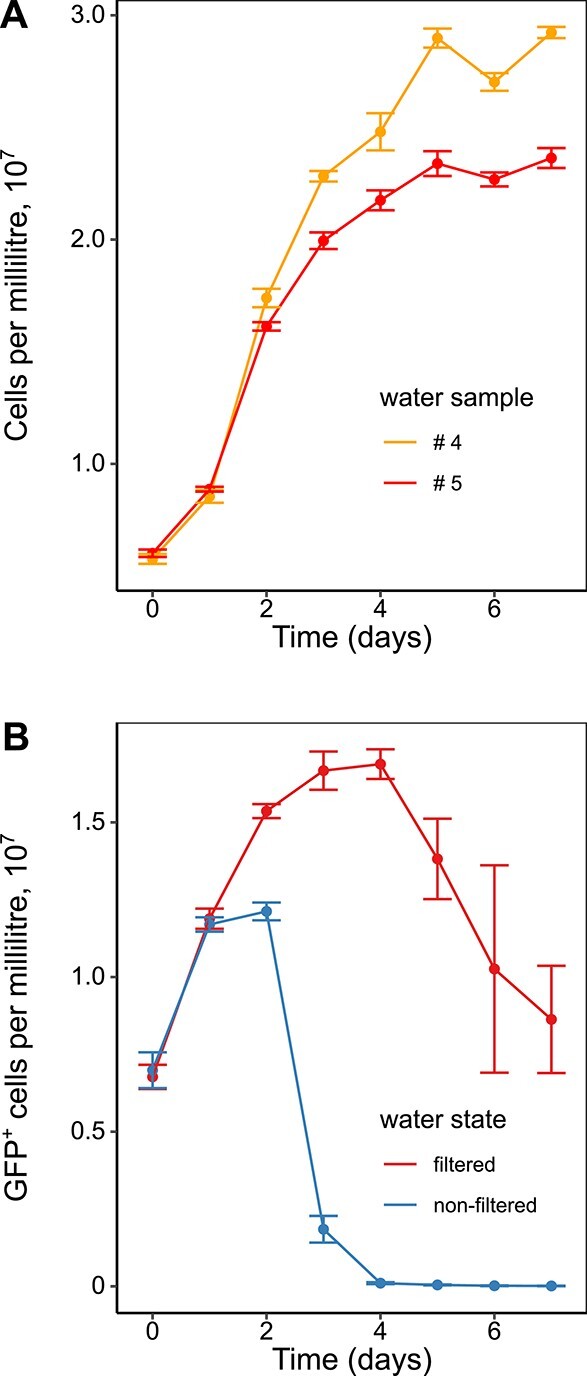
Growth of *E. coli* in lake water; (A) growth of *E. coli* in filtered lake water, assessed by flow cytometry as described in Materials and methods in two different samples of water (#4 and #5) as indicated; the error bars represent the standard error of the mean across three biological replicates; (B) growth of *E. coli* in filtered versus non-filtered water; for this experiment, the starting cultures of *E. coli* were transformed with an inducible plasmid to express GFP as a marker; colours represent filtered water and non-filtered water as indicated from the same water sample #5; the error bars represent the standard error of the mean across three biological replicates.

As illustrated by the control experiments, where *E. coli* cell counts were measured daily under these conditions by flow cytometry, the parental strain W3110 RpoS^+^ was able to grow in filtered lake water (Fig. 1A). Differences in growth observed between the two experimental repeats may be explained by the difference between the lake water samples. The sample collected at a higher ambient temperature apparently supported growth to a higher cell density, likely due to the increased levels of nutrients. To assess the pattern of *E. coli* cell number development in the presence of the autochthonous microbiota, we tested the growth of the same W3110 RpoS^+^ strain but transformed with an expression plasmid encoding GFP, which enabled us to distinguish and quantify *E. coli* cells by flow cytometry, even in the presence of other microorganisms. Incubation of this GFP-labelled parental *E. coli* strain in either filter-sterilized or non-filtered water from the same water sample showed that, after Day 2, *E. coli* cell numbers declined much more rapidly in the presence of the autochthonous microbiota (Fig. 1B). Although this experiment was performed in the same water sample #5 as one of the experiments with unlabelled *E. coli* (Fig.1A), the count of GFP-labelled *E. coli* cells was lower and subsequently decreased over time even during incubation in filtered water, which is likely explained by the burden of GFP expression on *E. coli* fitness.

The experiments with the RB-TnSeq library were performed with unlabelled *E. coli* cells to avoid such burden, and the development of cell numbers was not monitored, but the overall pattern of growth and survival of this library was assumed to follow the same general trends as observed for the parental strain in the control experiments. The RB-TnSeq library cultures were incubated in three water samples collected at different times (also different from the samples used in the control experiments). Cells were harvested after 2, 4, or 8 days of incubation, and the DNA was extracted for the subsequent PCR and library preparation. Following sequencing and barcode assignment, the fitness of each mutant was calculated as described in Wetmore *et al*. [11] (see also *Materials and methods* and Supplementary Table S4).

### RB-TnSeq reveals two major groups of differentially enriched mutations in lake water

**Figure 2:**
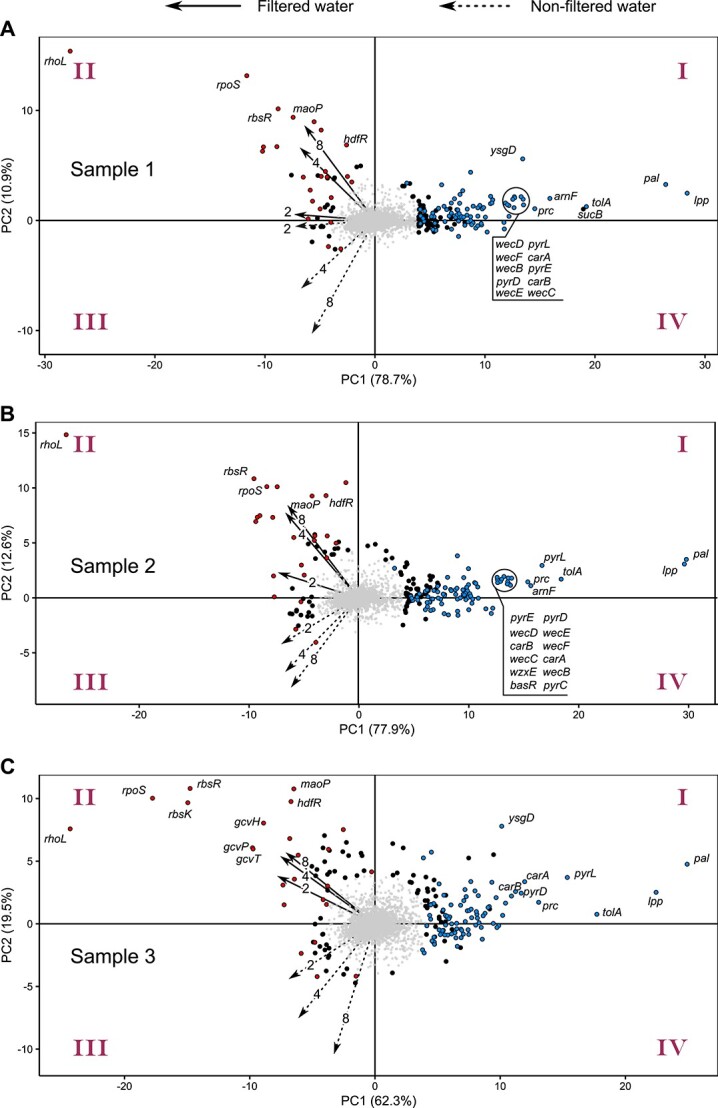
PCA applied to *E. coli* gene fitness data in lake water; PCA of changes in the abundance of individual gene mutations during incubation of *E. coli* in different indicated samples of filtered or non-filtered lake water #1(A), #2 (B), and #3 (C) (see Supplementary Table S2); the axes represent the first two principal components (PCs), which together account for >80% of the variability (Supplementary Fig. S3), with the quadrants of the plot being labelled; the % values in the brackets indicate the proportion of variability explained by each PC; arrows show projections of the gene fitness values from the original datasets (see Supplementary Table S4) for filtered (solid) and non-filtered (dashed) lake water, with time (days) of sampling being indicated; the effects of mutations on the gene fitness in filtered and non-filtered water (Supplementary Table S4) are thus reflected by their position along the corresponding arrows: the mutations in the Quadrants I and IV respectively reduce fitness in non-filtered and/or filtered water, whereas the mutations in the Quadrants II and III respectively increase fitness in filtered and/or non-filtered water; gene mutations with strongest fitness effects according to their Euclidean distance from the centre are shown as large dots (top 5%), or large dots with corresponding gene names (top 0.5%); gene mutations appearing in the top 5% in all three biological replicates are shown in red (increased fitness) or in blue (reduced fitness).

The majority of mutations in the RB-TnSeq library showed little or no change in frequency within the population, as expected. Nevertheless, we observed a set of mutants having reproducibly lower or higher fitness in filtered and/or non-filtered water (Supplementary Fig. S1 and Supplementary Table S4). To further characterize this dataset, we applied Principal Component Analysis (PCA) that is widely used in the analysis of multidimensional data [29]. This analysis revealed two major groups of mutants with large fitness effects, according to their Euclidean distance from the centre and their position relative to the vectors corresponding to the maximum variation within each constituting sample (Fig. 2 and Supplementary Fig. S2). In this representation, the fitness effects of individual mutations in filtered and non-filtered water correspond to their positions (positive or negative) along these vectors. Most mutants with the largest fitness effects were reproducibly selected between experiments (compare top 0.5% selected mutants in individual panels of Fig. 2).

The first large group of mutants is located primarily in the Quadrant I and partly in the Quadrant IV of the PCA plots and have high values of PC1 (highlighted in blue in Fig. 2), with its most prominent representatives being mutations in genes encoding components of the Tol-Pal system (*tolA*, *pal*, and *lpp*). This group represents mutants that have negative gene fitness values in non-filtered and filtered water (Supplementary Table S4) and thus have been strongly depleted. Nearly all of these gene mutations were detrimental in both filtered and non-filtered water, as can also be seen when their fitness effects are grouped using hierarchical clustering (Supplementary Fig. S1), suggesting similar selection under both conditions. Nevertheless, negative fitness effects were typically more pronounced in non-filtered water, presumably due to the competition and the predation by the autochthonous microbiota. Consistently, we observed no mutations with reproducible negative fitness effect specific to filtered water, which would have appeared at low positive values of PC1 in the Quadrant IV. Only a few mutants, most prominently *ysgD*, showed clear negative fitness effects in non-filtered water while being nearly neutral in filtered water (Fig. 2 and Supplementary Fig. S2).

The second prominent group of mutants is located in the Quadrant II of the PCA plots (highlighted in red in Fig. 2). These mutants show a fitness advantage (i.e. positive fitness values) in filtered water, but not in non-filtered water (Supplementary Fig. S1 and Supplementary Table S4). The strongest selection within this group between the samples was observed for the *rhoL*, *rpoS*, and *rbsR* genes.

In addition to these major groups, several mutations were specifically beneficial in non-filtered water (*glpK*, *glpF*; highlighted in red in the Quadrant III of Fig. 2) or in both filtered and non-filtered water (*iscR*, *tsaC*; Supplementary Fig. S2). However, their fitness effects were less pronounced than for the mutations in the two major groups.

This overall pattern of enrichment or depletion of gene mutations was highly reproducible between all three independent samples of the lake water, despite some sample-specific differences in the magnitude of gene fitness effects (Fig. 2, Supplementary Fig. S1, and Supplementary Table S4). These differences are likely explained by the nutrient variation between the water samples due to the different time of their collection (Supplementary Table S2), as already suggested by the moderate differences in *E. coli* growth (Fig. 1A).

### Cell envelope integrity, chemotaxis, ribonucleoside biosynthesis, and general stress response are important for growth or persistence in lake water

**Figure 3:**
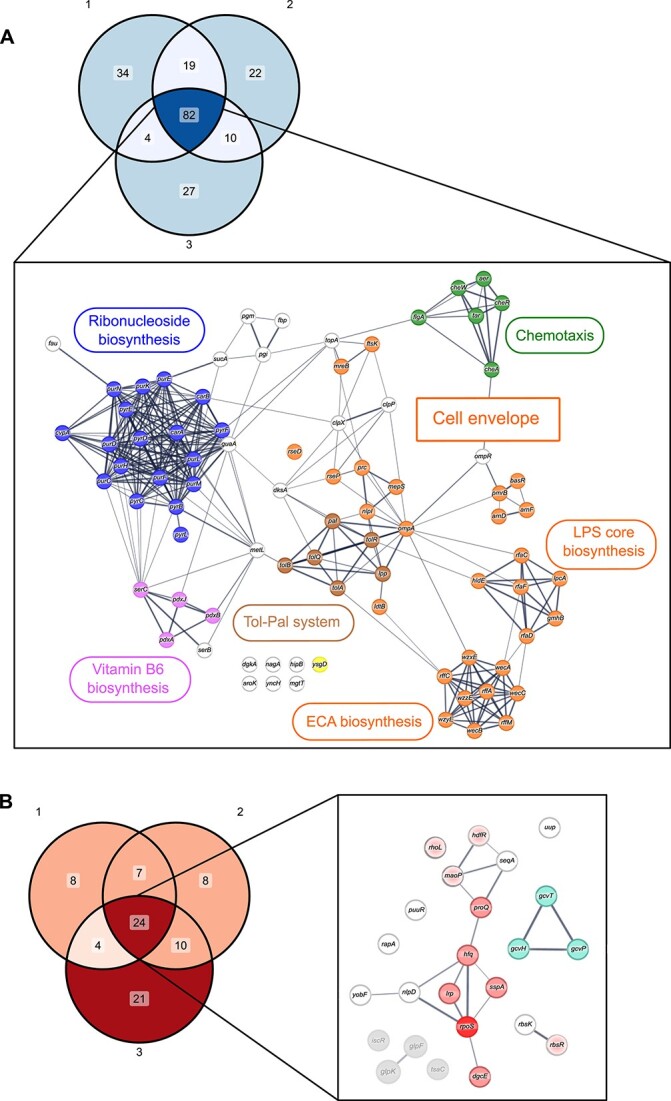
Functional clustering of gene mutations affecting *E. coli* fitness in lake water; (A) gene mutations that were reproducibly depleted in both filtered and non-filtered water (blue in Fig. 2; see also Supplementary Fig. S2) in three individual biological replicates, with the Euclidean distance cutoff of 5% (top); the STRING diagram (bottom) shows the clustering of genes common to all three replicates, with ribonucleoside biosynthesis highlighted in blue, vitamin B6 biosynthesis in purple, *ysgD* in yellow, chemotaxis-related genes in green, and cell envelope genes in orange (including the Tol-Pal system, shown in dark orange); the thickness of the lines indicates the strength of data support in STRING; (B) gene mutations that were reproducibly enriched in the filtered lake water (red in Fig. 2; see also Supplementary Fig. S2) in three individual biological replicates, with a Euclidean distance cutoff of 5% (left); the STRING diagram (right) shows the clustering of genes common to all three replicates; the glycine cleavage system is shown in turquoise; stress-response-related genes are highlighted in red (dark red for *rpoS*), with the newly described regulators of RpoS being shown in gradient red; gene mutations enriched only in the non-filtered water (Quadrant III of Fig. 2 and Supplementary Fig. S2) are indicated in grey.

To better understand the functionality of *E. coli* genes belonging to these two groups, we selected mutants that had the most prominent effect on fitness in all three experiments based on their Euclidean distance from the origin of the first two principal components (Supplementary Table S4), and then performed the enrichment analysis for the top 5% genes common across all three replicates using the STRING database [26] (Fig. 3).

Most genes in the group of mutants with reproducibly negative fitness effects in non-filtered and filtered water map to several well-defined functional clusters (Fig. 3A). The strongest depletion was observed for mutations in the genes involved in the biogenesis of the bacterial cell envelope, primarily the outer membrane (Fig. 3A, orange). In addition to already mentioned mutations in the Tol-Pal system, these included the major outer membrane protein OmpA, and the biosynthetic pathways of the enterobacterial common antigen (ECA) and lipopolysaccharide (LPS). All of these mutations could reduce the protective function of the outer membrane, making *E. coli* more sensitive to toxins and antibiotics [30–32]. This group of mutations might additionally lead to leakage of the periplasmic content into the medium [30, 31], which might be particularly detrimental in the hypoosmotic and low-nutrient lake water that might contain toxic substances.

Another cluster of depleted mutants consists of chemotaxis genes (Fig. 3A, green, and Supplementary Fig. S2), suggesting that the ability of motile *E. coli* to follow nutrient and/or oxygen gradients, and possibly to avoid high concentrations of toxic substances [33, 34], provides fitness benefit in lake water. Negative fitness impact was also observed for clusters of mutations in biosynthetic pathways for ribonucleosides and vitamin B6.

A pronounced fitness defect in non-filtered water was also observed for the mutation in a gene encoding a small protein of unknown function YsgD [35] (Fig. 2). The *ysgD* gene is located directly upstream of the Ni^2+^/Co^2+^/Mg^2+^ transporter *corA*, and might be involved in the transcriptional regulation of this transporter [36]. This gene may be specifically important for growth and/or survival in the presence of the autochthonous microbiota, but the underlying mechanism remains to be elucidated. Similar but weaker specific negative fitness effects in non-filtered water have been observed for mutations in genes encoding the DNA topoisomerase I (TopA) [37] and the protease RseP, which regulates the cell envelope stress response [38] (Supplementary Fig. S2).

Within the second group of mutants, those showing fitness advantage in filtered water (Fig. 3B), many of the characterized genes mapped to *E. coli* stress response pathways. Besides the prominent enrichment of *rpoS*, these included genes encoding the RNA-binding proteins Hfq [39] and ProQ [40], the global transcriptional regulators Lrp [41] and SspA [42], and the major diguanylate cyclase DgcE that controls *E. coli* lifestyle transition to the biofilm state [14, 43] (Fig. 3B, red). A very similar pattern of enrichment to that of *rpoS* was also observed for mutations in several genes with apparently unrelated functions, including three poorly annotated genes *rhoL*, *hdfR*, and *maoP*, and also in *rbsR* which encodes the repressor of ribose catabolism [44–47]. Enrichment was also observed for several genes of the glycine cleavage system (*gcvH*, *gcvP*, and *gcvT*). This group of proteins catalyses the oxidation of glycine to carbon dioxide and ammonia, yielding a methylene group accepted by tetrahydrofolate and one NADH [48].

Two mutants (*glpK* and *glpF*), encoding components of the glycerol utilization pathway [49], showed a specific fitness advantage in non-filtered water (Fig. 3B, grey, and Supplementary Fig. S2). Several other mutants showed increased frequency in both filtered and non-filtered water (Supplementary Fig. S2), including mutations in genes that encode the threonylcarbamoyl-AMP synthase TsaC [50], the iron–sulphur cluster containing transcription factor IscR [51], and the negative regulator of replication SeqA [52].

### Mutant clustering enables identification of novel components of the general stress-response pathway of *E. coli*

**Figure 4:**
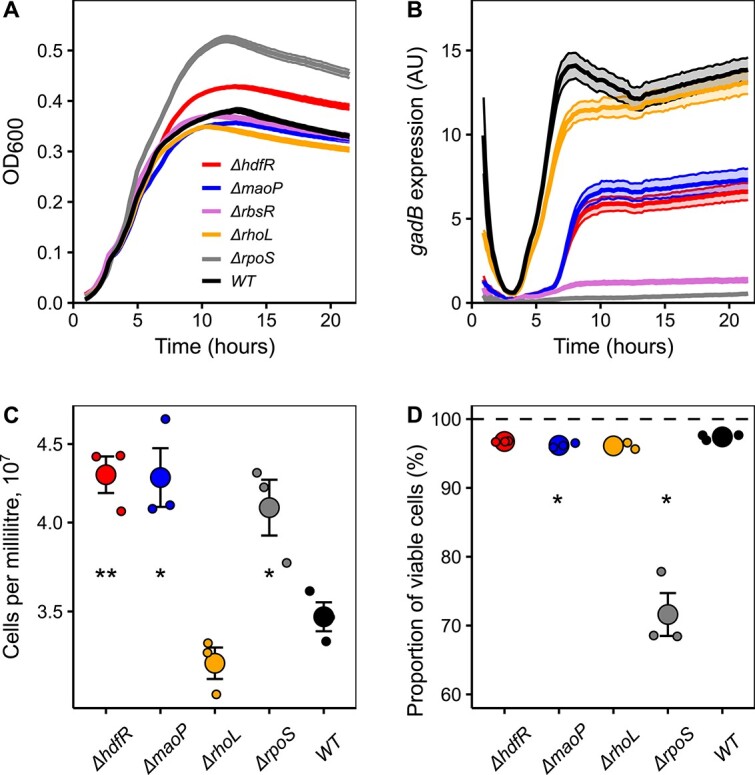
Effects of growth-benefiting mutations on RpoS activity and cell viability; (A) growth of the indicated gene knockout strains in a rich medium; the OD_600_ was monitored in a plate reader during growth in TB medium; a representative experiment is shown with the average of three technical replicates; the shading shows the standard error of the mean; (B) activity of the RpoS-dependent *gadB* promoter, assessed by GFP reporter, in the indicated gene knockout strains; GFP fluorescence was monitored during culture growth in TB medium in a plate reader and normalized to cell density (OD_600_; Fig. 4A), and expressed in AUs; a representative experiment is shown with the average of three technical replicates; the shading shows the standard error of the mean; (C) total cell counts in the populations of the indicated mutants, as accessed by flow cytometry, after 8 days of incubation in filtered water sample #4; the values were measured in three biological replicates, three technical replicates each; (D) percentage of viable cells in the populations of the indicated mutants, as accessed by SYTOX Orange staining, after 8 days of incubation in filtered water sample #4; the values were measured in three biological replicates, three technical replicates each; in panels C and D, the small dots represent the average values of each biological replicate, whereas the large dots represent the average values of three biological replicates; the error bars indicate the standard error of the mean; *P* values for the comparison of each strain to the wild type strain were calculated using a Welch’s *t*-test (*, *P* value < .05; **, *P* value < .01).

Given the reproducible clustering of several poorly annotated genes with *rpoS* among *E. coli* mutants that were enriched during growth in filtered water, we hypothesized that mutations in these genes might have common impact on RpoS activity. Indeed, *E. coli* strains deleted for *rhoL*, *hdfR*, *maoP*, or *rbsR* exhibited reduced expression of the RpoS-dependent *gadB* promoter [5] when grown in the nutrient-rich TB medium (Fig. 4A and B). The extent of such reduction was gene-dependent, with the deletion of *rhoL* having only a transient impact at the entry into the stationary phase, the deletions of *hdfR* and *maoP* having almost identical intermediate impact, and the deletion of *rbsR* even nearly abolishing the *gadB* promoter activity. These results confirm the importance of these genes for the activation of the general stress response in *E. coli* in addition to their other established or proposed functions [44–47].

To better understand the reasons for the enrichment of the *rpoS* and other mutations in filtered but not in non-filtered water, we directly assessed the growth and viability of these mutants. Consistent with our RB-TnSeq analysis, Δ*hdfR*, Δ*maoP*, and Δ*rpoS* strains reached significantly higher cell counts than the wild type when grown individually in filtered lake water (Fig. 4C). Two of these mutants, Δ*hdfR* and Δ*rpoS*, also grew to the higher cell density in the rich TB medium (Fig. 4A). No significant difference in growth from the wild type was observed for the Δ*rhoL* strain, possibly consistent with the smallest impact of this deletion on RpoS activity (Fig. 4B).

We further determined the viability of the Δ*rpoS* strain, as well as of Δ*hdfR*, Δ*maoP*, and Δ*rhoL* strains that show intermediate levels of the RpoS activity, after 8-day long incubation in filtered lake water using live-dead staining with SYTOX Orange (Fig. 4D). The viability of the Δ*rpoS* strain was strongly compromised compared to the wild type, in agreement with previous studies [3, 6, 7, 53], whereas the viability of the other tested strains was not significantly different (Δ*hdfR* and Δ*rhoL*) or only weakly reduced (Δ*maoP*). Thus, an intermediate level of the RpoS activity observed in these strains might balance the growth-survival trade-off in lake water.

Complementary to the live-dead staining, we assessed the viability of cells in the same Δ*rpoS* and Δ*rhoL* cultures using counting of colony-forming units (Supplementary Fig. S4). Although the overall fraction of viable cells determined by this method was expectedly lower than determined by SYTOX Orange staining [54], the relative differences in the viability were consistent between the assays. Again, we observed the reduced viability of the Δ*rpoS* strain compared to the wild type, whereas the Δ*rhoL* strain showed higher viability than the Δ*rpoS* strain.

## Discussion

*E. coli* and other enteric bacteria are known to persist and proliferate extraintestinally for extended periods of time, which might play an important role in epidemiology [1, 8, 55], but the genetic determinants of such persistence have not been systematically characterized to date. Here, we have conducted a comprehensive whole-genome screening of *E. coli* growth and survival in lake water using transposon mutagenesis. Although our study was conducted for the derivative of the laboratory K-12 strain of *E. coli*, enabling us to benefit from its excellent genome annotation, we used the RpoS^+^ lineage, which serves as a model for biofilm formation and environmental stress response [14–18]. Furthermore, the identified genetic determinants of growth and persistence in lake water map to general cellular and regulatory functions of *E. coli,* and their importance is likely to be conserved among commensal or pathogenic *E. coli* strains.

As expected, the majority of gene mutations identified in our screen were detrimental to the growth and persistence of *E. coli*. A prominent cluster of such mutations mapped to the genes required for cell envelope integrity and the protective function of the outer membrane, including the Tol-Pal system, the major outer membrane protein OmpA, and the biosynthesis of the ECA and LPS, potentially making *E. coli* more susceptible to toxins and antibiotics [30–32] and lead to leakage of periplasmic content into the medium [30, 31]. Thus, the intact cell envelope appears to be a crucial factor for *E. coli* survival in the hypoosmotic lake water environment. Notably, this protective function of the cell envelope might be particularly important for the K-12 lineages of *E. coli*, including W3110, that are not able to produce the protective O-antigen [56] and are therefore more sensitive to the cell-envelope stress.

Another cluster of mutations with negative effects on *E. coli* fitness involved the nucleoside biosynthetic pathway, especially its pyrimidine biosynthetic part. Mutants in the biosynthetic pathway of vitamin B6 which is structurally close to pyrimidines were also depleted. Lake water is unlikely to contain copious amounts of dissolved nucleosides or vitamins, and the bacteria have to rely on their own machinery for their biosynthesis. Potentially consistent with this, the Δ*rbsR* strain, which is expected to de-repress the de novo biosynthesis of purine nucleotides [57], showed a fitness advantage in lake water.

Negative impact on fitness was also observed for mutations in most chemotaxis genes of *E. coli*. Chemotaxis of motile bacteria is known to be important for nutrient acquisition in natural environments [33, 34] and it has previously been demonstrated to provide growth fitness benefit under carbon-limited growth conditions [58]. The chemotaxis pathway of *E. coli* and other bacteria is also known to mediate accumulation towards optimal oxygen levels, and the depletion of the mutation in *aer* encoding *E. coli* aerotaxis receptor [59] indicates the importance of such aerotaxis for *E. coli* growth in lake water. No depletion of mutations in flagellar genes that are required for motility and chemotaxis was observed in our study, possibly because the detrimental effect of the inability to perform chemotaxis is compensated in this case by the elimination of the high cost of flagellar production [58].

All of these mutants had negative fitness effects in both filtered and non-filtered water, although their impact in non-filtered water was typically more pronounced. This was expected, because reduced growth or decreased cell survival of mutants in filtered water is likely to decrease their relative frequencies in the population in the presence of the autochthonous microbiota, and these fitness defects may be even further enhanced by the additional competition and predation. Only a handful of mutations caused selective reduction of fitness in non-filtered water, affecting a small protein of unknown function YsgD [35, 36], the DNA topoisomerase I (TopA) [37], and the membrane protease RseP that induces the σ^E^-dependent stress response [38]. Why these mutations specifically reduce survival in the presence of the autochthonous microbiota but not (or much less) in its absence remains to be understood.

In addition to these mutations with the negative impact on fitness, we also identified a group of mutations that were beneficial for *E. coli* to proliferate or survive in lake water, primarily in the absence of the autochthonous microbiota. We observed that most of these apparently beneficial mutations impaired the general stress response by disrupting *rpoS* or reducing RpoS activity. Although the importance of an intact and active RpoS has been demonstrated in both seawater [3] and soil [7], studies have shown that complete or partial loss of *rpoS* function results in lower viability and higher susceptibility to predation by protozoa in soil [3, 6, 55, 60, 61]. Despite this, environmental populations of *E. coli* and *Salmonella* have a high frequency of polymorphisms in *rpoS* [62] including complete loss of its function [4]. Moreover, the emergence of loss-of-function *rpoS* mutations has been observed in laboratory evolution experiments during growth on poor media or on non-preferred carbon source [62–64]. It has been proposed that these mutants have a growth advantage due to the repression of the *rpoS* regulon, which enables them to upregulate the metabolic σ^70^-dependent genes, although this reduces their ability of stress response [62, 64, 65]. This suggests a duality of the RpoS function, with its low activity being advantageous for growth in low-nutrient environments but disadvantageous for stress resistance and survival [9].

Consistent with such dualism of the RpoS function, we observed that, despite their better growth, the viability of the Δ*rpoS* strain in filtered lake water was severely compromised. In contrast, the viability of the other selected mutants with intermediate activity of RpoS was less different from the wild-type strain. We hypothesize that these strains may better balance the competitive advantage in the low-nutrient environment with the ability to withstand other stresses, due to the reduced but not abolished activity of RpoS.

Although all mutations with negative fitness effects in filtered water also had reduced fitness in the presence of the autochthonous microbiota, none of the mutations that reduced RpoS activity were enriched in the presence of the autochthonous microbiota. This may be due to the increased susceptibility of the RpoS-deficient cells to predation [7] or their reduced competiveness with the autochthonous microbiota, thus highlighting another aspect of the RpoS duality.

The observed co-enrichment of mutations in several poorly characterized *E. coli* genes (*hdfR*, *maoP*, *rbsR,* and *rhoL*) with *rpoS* enabled us to assign them a novel function in the RpoS regulation. Further co-enriched mutations in other stress-response genes might have also been enriched because of their impact on the RpoS activity. Given that the regulation of RpoS in *E. coli* has already been described in great detail [5, 17, 66], our discovery of several additional regulators was unexpected and demonstrates the complexity of the general stress response in bacteria. The exact mechanisms of the effects on the RpoS activity and its relation to the other established functions of these regulators [5, 40–42, 44–47] remain to be investigated.

## Study limitations

The *E. coli* K-12 W3110 RpoS^+^ strain used in this study is a derivative of the laboratory strain with the restored RpoS activity. This strain has been used in studies of stress-response-related functions and has a high quality genome annotation, which is beneficial for the creation and analysis of a comprehensive transposon mutant library. But although the general cellular functions that have been shown to affect the fitness of this strain in lake water are highly conserved, other *E. coli* isolates are likely to exhibit strain-specific differences in fitness that need to be characterized.

## Supplementary Material

Supplementary_Figure_S1_wrae096

Supplementary_Figure_S2_wrae096

Supplementary_Figure_S3_wrae096

Supplementary_Figure_S4_wrae096

Supplementary_Table_S1_wrae096

Supplementary_Table_S2_wrae096

Supplementary_Table_S3_wrae096

Supplementary_Table_S4_wrae096

## Data Availability

The high-throughput sequencing data are available at NCBI BioProject database under the accession numbers PRJNA1043681 (lake water experiment) and PRJNA1073534 (Tn5 library annotation). The scripts are deposited at GitHub (https://github.com/NataliyaTeteneva/Tn5_library_analysis). The full table of the gene fitness data is available as the Supplementary Table S4. The other datasets generated and analysed during the current study are available from the authors on request.

## References

[1] van Elsas JD , Semenov AV, Costa R et al. Survival of *Escherichia coli* in the environment: fundamental and public health aspects. ISME *J* 2011;5:173–83. 10.1038/ismej.2010.80 20574458 PMC3105702

[2] Solo-Gabriele HM , Wolfert MA, Desmarais TR et al. Sources of *Escherichia coli* in a coastal subtropical environment. Appl Environ Microbio*l* 2000;66:230–7. 10.1128/AEM.66.1.230-237.2000 10618229 PMC91811

[3] Rozen Y , Belkin S. Survival of enteric bacteria in seawater. FEMS Microbiol Re*v* 2001;25:513–29. 10.1111/j.1574-6976.2001.tb00589.x 11742689

[4] Chiang SM , Dong T, Edge TA et al. Phenotypic diversity caused by differential RpoS activity among environmental *Escherichia coli* isolates. Appl Environ Microbio*l* 2011;77:7915–23. 10.1128/AEM.05274-11 21948830 PMC3209002

[5] Hengge R . Stationary-phase gene regulation in *Escherichia coli*. EcoSal Plu*s* 2011;4. 10.1128/ecosalplus.5.6.3 26442507

[6] Somorin Y , Abram F, Brennan F et al. The general stress response is conserved in long-term soil-persistent strains of *Escherichia coli*. Appl Environ Microbio*l* 2016;82:4628–40. 10.1128/AEM.01175-16 27235429 PMC4984288

[7] Somorin Y , Bouchard G, Gallagher J et al. Roles for RpoS in survival of *Escherichia coli* during protozoan predation and in reduced moisture conditions highlight its importance in soil environments. FEMS Microbiol Let*t* 2017;364:fnx198. 10.1093/femsle/fnx198 28967947

[8] Touchon M , Perrin A, de Sousa JAM et al. Phylogenetic background and habitat drive the genetic diversification of *Escherichia coli*. PLoS Gene*t* 2020;16:e1008866. 10.1371/journal.pgen.1008866 32530914 PMC7314097

[9] Valencia EY , Barros JP, Ferenci T et al. A broad continuum of *E. coli* traits in nature associated with the trade-off between self-preservation and nutritional competence. Microb Eco*l* 2022;83:68–82. 10.1007/s00248-021-01751-6 33846820

[10] Rumball NA , Alm EW, McLellan SL. Genetic determinants of *Escherichia coli* survival in beach sand. Appl Environ Microbio*l* 2023;89:e0142322. 10.1128/aem.01423-22 36515536 PMC9888298

[11] Wetmore KM , Price MN, Waters RJ et al. Rapid quantification of mutant fitness in diverse bacteria by sequencing randomly bar-coded transposons. mBi*o* 2015;6:e00306–15. 10.1128/mBio.00306-15 25968644 PMC4436071

[12] Warr AR , Hubbard TP, Munera D et al. Transposon-insertion sequencing screens unveil requirements for EHEC growth and intestinal colonization. PLoS Patho*g* 2019;15:e1007652. 10.1371/journal.ppat.1007652 31404118 PMC6705877

[13] Hayashi K , Morooka N, Yamamoto Y et al. Highly accurate genome sequences of *Escherichia coli* K-12 strains MG1655 and W3110. Mol Syst Bio*l* 2006;2:0007. 10.1038/msb4100049 PMC1681481 16738553

[14] Serra DO , Richter AM, Klauck G et al. Microanatomy at cellular resolution and spatial order of physiological differentiation in a bacterial biofilm. mBi*o* 2013;4:e00103–13. 10.1128/mBio.00103-13 23512962 PMC3604763

[15] Besharova O , Suchanek VM, Hartmann R et al. Diversification of gene expression during formation of static submerged biofilms by *Escherichia coli*. Front Microbio*l* 2016;7:1568. 10.3389/fmicb.2016.01568 27761132 PMC5050211

[16] Teteneva NA , Mart'yanov SV, Esteban-Lopez M et al. Multiple drug-induced stress responses inhibit formation of *Escherichia coli* biofilms. Appl Environ Microbio*l* 2020;86:e01113–20. 10.1128/AEM.01113-20 32826218 PMC7580552

[17] Pesavento C , Becker G, Sommerfeldt N et al. Inverse regulatory coordination of motility and curli-mediated adhesion in *Escherichia coli*. Genes De*v* 2008;22:2434–46. 10.1101/gad.475808 18765794 PMC2532929

[18] Cordisco E , Zanor MI, Moreno DM et al. Selective inhibition of the amyloid matrix of *Escherichia coli* biofilms by a bifunctional microbial metabolite. NPJ Biofilms Microbiome*s* 2023;9:81. 10.1038/s41522-023-00449-6 37857690 PMC10587114

[19] Datsenko KA , Wanner BL. One-step inactivation of chromosomal genes in *Escherichia coli* K-12 using PCR products. Proc Natl Acad Sci U S *A* 2000;97:6640–5. 10.1073/pnas.120163297 10829079 PMC18686

[20] Jensen SI , Lennen RM, Herrgard MJ et al. Seven gene deletions in seven days: fast generation of *Escherichia coli* strains tolerant to acetate and osmotic stress. Sci Re*p* 2015;5:17874. 10.1038/srep17874 26643270 PMC4672327

[21] Amann E , Ochs B, Abel KJ. Tightly regulated *tac* promoter vectors useful for the expression of unfused and fused proteins in *Escherichia coli*. Gen*e* 1988;69:301–15. 10.1016/0378-1119(88)90440-4 3069586

[22] Cock PJ , Antao T, Chang JT et al. Biopython: freely available python tools for computational molecular biology and bioinformatics. Bioinformatic*s* 2009;25:1422–3. 10.1093/bioinformatics/btp163 19304878 PMC2682512

[23] Camacho C , Coulouris G, Avagyan V et al. BLAST+: architecture and applications. BMC Bioinformatic*s* 2009;10:421. 10.1186/1471-2105-10-421 20003500 PMC2803857

[24] Andrews S , Krueger F, Segonds-Pichon A et al. FastQC: A Quality Control Tool for High Throughput Sequence Data. Babraham, UK: Babraham Institute, 2010

[25] Ewels P , Magnusson M, Lundin S et al. MultiQC: summarize analysis results for multiple tools and samples in a single report. Bioinformatic*s* 2016;32:3047–8. 10.1093/bioinformatics/btw354 27312411 PMC5039924

[26] Szklarczyk D , Kirsch R, Koutrouli M et al. The STRING database in 2023: protein-protein association networks and functional enrichment analyses for any sequenced genome of interest. Nucleic Acids Re*s* 2023;51:D638–46. 10.1093/nar/gkac1000 36370105 PMC9825434

[27] Zaslaver A , Bren A, Ronen M et al. A comprehensive library of fluorescent transcriptional reporters for *Escherichia coli*. Nat Method*s* 2006;3:623–8. 10.1038/nmeth895 16862137

[28] Schindelin J , Arganda-Carreras I, Frise E et al. Fiji: an open-source platform for biological-image analysis. Nat Method*s* 2012;9:676–82. 10.1038/nmeth.2019 22743772 PMC3855844

[29] Jolliffe IT , Cadima J. Principal component analysis: a review and recent developments. Philos Trans A Math Phys Eng Sc*i* 2016;374:20150202 26953178 10.1098/rsta.2015.0202 PMC4792409

[30] Asmar AT , Collet JF. Lpp, the Braun lipoprotein, turns 50-major achievements and remaining issues. FEMS Microbiol Let*t* 2018;365:fny199. 10.1093/femsle/fny199 30107563

[31] Cascales E , Bernadac A, Gavioli M et al. Pal lipoprotein of *Escherichia coli* plays a major role in outer membrane integrity. J Bacterio*l* 2002;184:754–9. 10.1128/JB.184.3.754-759.2002 11790745 PMC139529

[32] Rai AK , Mitchell AM. Enterobacterial common antigen: synthesis and function of an enigmatic molecule. mBi*o* 2020;11:e01914–20. 10.1128/mBio.01914-20 32788387 PMC7439462

[33] Colin R , Ni B, Laganenka L et al. Multiple functions of flagellar motility and chemotaxis in bacterial physiology. FEMS Microbiol Re*v* 2021;45:fuab038. 10.1093/femsre/fuab038 PMC8632791 34227665

[34] Keegstra JM , Carrara F, Stocker R. The ecological roles of bacterial chemotaxis. Nat Rev Microbiol*.* 2022;20:491–504. 10.1038/s41579-022-00709-w 35292761

[35] Weaver J , Mohammad F, Buskirk AR et al. Identifying small proteins by ribosome profiling with stalled initiation complexes. mBi*o* 2019;10:e02819–8. 10.1128/mBio.02819-18 30837344 PMC6401488

[36] Oh E , Becker AH, Sandikci A et al. Selective ribosome profiling reveals the cotranslational chaperone action of trigger factor *in vivo*. Cel*l* 2011;147:1295–308. 10.1016/j.cell.2011.10.044 22153074 PMC3277850

[37] Brochu J , Breton EV, Drolet M. Supercoiling, R-loops, replication and the functions of bacterial type 1A topoisomerases. Genes (Basel*)* 2020;11:249. 10.3390/genes11030249 32120891 PMC7140829

[38] Alba BM , Leeds JA, Onufryk C et al. DegS and YaeL participate sequentially in the cleavage of RseA to activate the σ^E^-dependent extracytoplasmic stress response. Genes De*v* 2002;16:2156–68. 10.1101/gad.1008902 12183369 PMC186436

[39] Brennan RG , Link TM. Hfq structure, function and ligand binding. Curr Opin Microbio*l* 2007;10:125–33. 10.1016/j.mib.2007.03.015 17395525

[40] Holmqvist E , Li L, Bischler T et al. Global maps of ProQ binding *in vivo* reveal target recognition via RNA structure and stability control at mRNA 3′ ends. Mol Cel*l* 2018;70:971–82.e6. 10.1016/j.molcel.2018.04.017 29804828

[41] Cho BK , Barrett CL, Knight EM et al. Genome-scale reconstruction of the Lrp regulatory network in *Escherichia coli*. Proc Natl Acad Sci U S *A* 2008;105:19462–7. 10.1073/pnas.0807227105 19052235 PMC2614783

[42] Hansen AM , Qiu Y, Yeh N et al. SspA is required for acid resistance in stationary phase by downregulation of H-NS in *Escherichia coli*. Mol Microbio*l* 2005;56:719–34. 10.1111/j.1365-2958.2005.04567.x 15819627

[43] Hengge R . Principles of c-di-GMP signalling in bacteria. Nat Rev Microbio*l* 2009;7:263–73. 10.1038/nrmicro2109 19287449

[44] Ko M , Park C. H-NS-dependent regulation of flagellar synthesis is mediated by a LysR family protein. J Bacterio*l* 2000;182:4670–2. 10.1128/JB.182.16.4670-4672.2000 10913108 PMC94646

[45] Valens M , Thiel A, Boccard F. The MaoP/*maoS* site-specific system organizes the *ori* region of the *E. coli* chromosome into a macrodomain. PLoS Gene*t* 2016;12:e1006309. 10.1371/journal.pgen.1006309 27627105 PMC5023128

[46] Matsumoto Y , Shigesada K, Hirano M et al. Autogenous regulation of the gene for transcription termination factor *rho* in *Escherichia coli*: localization and function of its attenuators. J Bacterio*l* 1986;166:945–58. 10.1128/jb.166.3.945-958.1986 2423505 PMC215217

[47] Mauzy CA , Hermodson MA. Structural and functional analyses of the repressor, RbsR, of the ribose operon of *Escherichia coli*. Protein Sc*i* 1992;1:831–42. 10.1002/pro.5560010701 1304369 PMC2142159

[48] Kikuchi G , Motokawa Y, Yoshida T et al. Glycine cleavage system: reaction mechanism, physiological significance, and hyperglycinemia. Proc Jpn Acad Ser B Phys Biol Sc*i* 2008;84:246–63. 10.2183/pjab.84.246 PMC3666648 18941301

[49] Voegele RT , Sweet GD, Boos W. Glycerol kinase of *Escherichia coli* is activated by interaction with the glycerol facilitator. J Bacterio*l* 1993;175:1087–94. 10.1128/jb.175.4.1087-1094.1993 8432702 PMC193024

[50] Deutsch C , El Yacoubi B, de Crecy-Lagard V et al. Biosynthesis of threonylcarbamoyl adenosine (t6A), a universal tRNA nucleoside. J Biol Che*m* 2012;287:13666–73. 10.1074/jbc.M112.344028 22378793 PMC3340167

[51] Santos JA , Pereira PJ, Macedo-Ribeiro S. What a difference a cluster makes: the multifaceted roles of IscR in gene regulation and DNA recognition. Biochim Biophys Act*a* 2015;1854:1101–12. 10.1016/j.bbapap.2015.01.010 25641558

[52] Waldminghaus T , Skarstad K. The *Escherichia coli* SeqA protein. Plasmi*d* 2009;61:141–50. 10.1016/j.plasmid.2009.02.004 19254745

[53] Munro PM , Flatau GN, Clement RL et al. Influence of the RpoS (KatF) sigma factor on maintenance of viability and culturability of *Escherichia coli* and *Salmonella typhimurium* in seawater. Appl Environ Microbio*l* 1995;61:1853–8. 10.1128/aem.61.5.1853-1858.1995 7646022 PMC167447

[54] Kumar SS , Ghosh AR. Assessment of bacterial viability: a comprehensive review on recent advances and challenges. Microbiology 2019;165:593–610. 10.1099/mic.0.000786 30843781

[55] Ishii S , Sadowsky MJ. *Escherichia coli* in the environment: implications for water quality and human health. Microbes Enviro*n* 2008;23:101–8. 10.1264/jsme2.23.101 21558695

[56] Liu D , Reeves PR. *Escherichia coli* K12 regains its O antigen. Microbiology 1994;140:49–57. 10.1099/13500872-140-1-49 7512872

[57] Shimada T , Kori A, Ishihama A. Involvement of the ribose operon repressor RbsR in regulation of purine nucleotide synthesis in *Escherichia coli*. FEMS Microbiol Let*t* 2013;344:159–65. 10.1111/1574-6968.12172 23651393

[58] Ni B , Colin R, Link H et al. Growth-rate dependent resource investment in bacterial motile behavior quantitatively follows potential benefit of chemotaxis. Proc Natl Acad Sci U S *A* 2020;117:595–601. 10.1073/pnas.1910849117 31871173 PMC6955288

[59] Taylor BL . Aer on the inside looking out: paradigm for a PAS-HAMP role in sensing oxygen, redox and energy. Mol Microbio*l* 2007;65:1415–24. 10.1111/j.1365-2958.2007.05889.x 17824925 PMC2586974

[60] Brennan FP , Grant J, Botting CH et al. Insights into the low-temperature adaptation and nutritional flexibility of a soil-persistent *Escherichia coli*. FEMS Microbiol Eco*l* 2013;84:75–85. 10.1111/1574-6941.12038 23134365

[61] Jang J , Hur HG, Sadowsky MJ et al. Environmental *Escherichia coli:* ecology and public health implications - a review. J Appl Microbio*l* 2017;123:570–81. 10.1111/jam.13468 28383815

[62] Ferenci T . What is driving the acquisition of *mutS* and *rpoS* polymorphisms in *Escherichia coli*? Trends Microbio*l* 2003;11:457–61. 10.1016/j.tim.2003.08.003 14557028

[63] Dong T , Chiang SM, Joyce C et al. Polymorphism and selection of *rpoS* in pathogenic *Escherichia coli*. BMC Microbio*l* 2009;9:118. 10.1186/1471-2180-9-118 19493358 PMC2700278

[64] Notley-McRobb L , King T, Ferenci T. *rpoS* mutations and loss of general stress resistance in *Escherichia coli* populations as a consequence of conflict between competing stress responses. J Bacterio*l* 2002;184:806–11. 10.1128/JB.184.3.806-811.2002 11790751 PMC139526

[65] Finkel SE . Long-term survival during stationary phase: evolution and the GASP phenotype. Nat Rev Microbiol 2006;4:113–20. 10.1038/nrmicro1340 16415927

[66] Battesti A , Majdalani N, Gottesman S. The RpoS-mediated general stress response in *Escherichia coli*. Ann Rev Microbio*l* 2011;65:189–213. 10.1146/annurev-micro-090110-102946 21639793 PMC7356644

