# Supplementary material for: Genome-wide screen of genetic determinants that govern Escherichia coli growth and persistence in lake water
Source: ISME J. 2024 Jun 14;18(1):wrae096. doi: 10.1093/ismejo/wrae096 (PMC11188689; doi:10.1093/ismejo/wrae096)
Supplement: Supplementary_Figure_S1_wrae096 [file supplementary_figure_s1_wrae096.pdf]

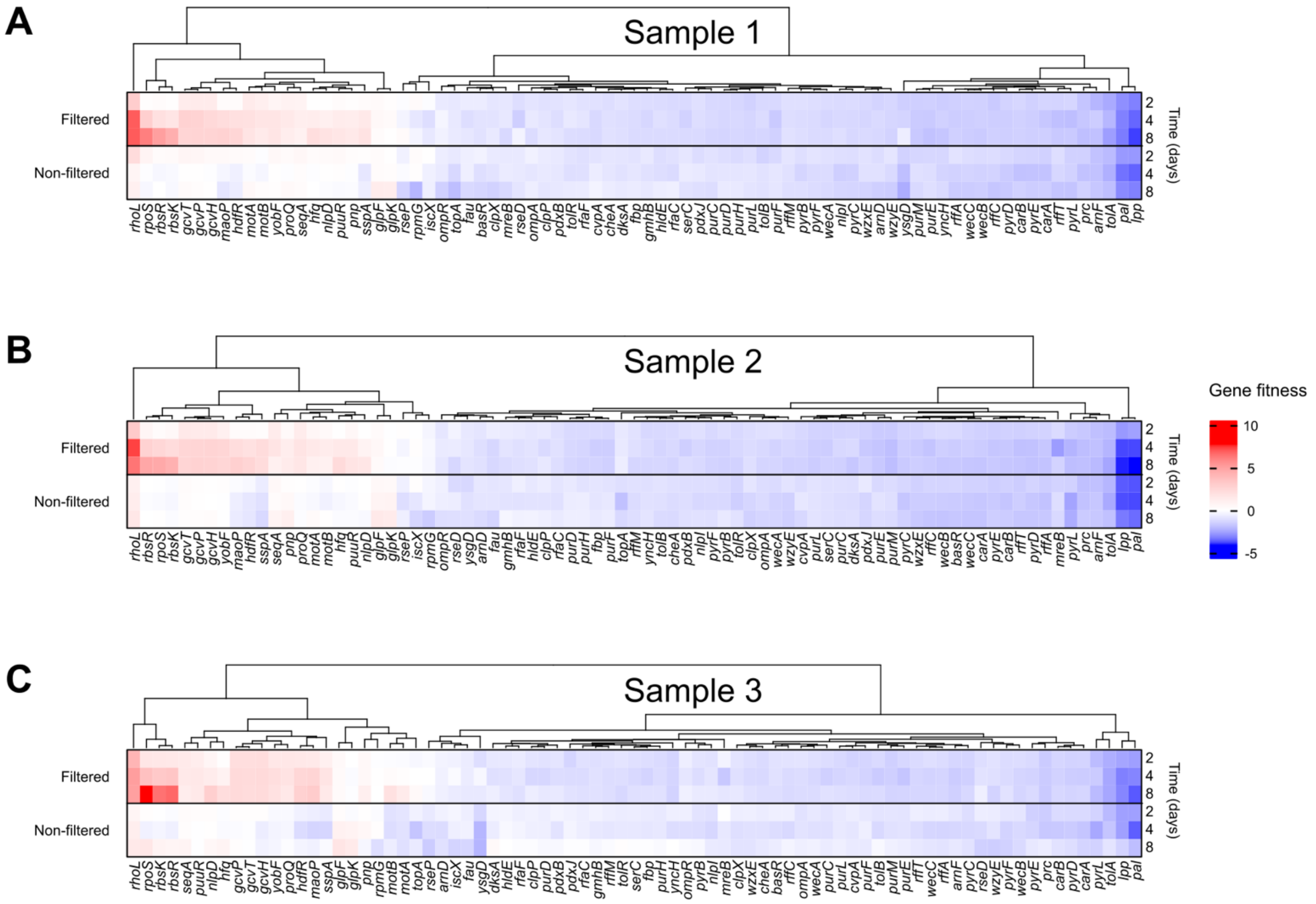

Supplementary Figure S1. Hierarchical clustering of RB-TnSeq gene fitness data.

Clustering is shown for water samples #1 (A), #2 (B), and #3 (C). Only mutations that affect fitness (with the absolute values of gene fitness greater than 1 in any of the samples) are shown. Clusters with similar patterns are indicated by the dendrogram branches at the top of each heatmap. Red colour represents increased fitness value and blue represents decreased fitness value, whereas the intensity of both colours reflects the absolute value of fitness. The plots were generated using R package ComplexHeatmap.
