# Supplementary material for: Genome-wide screen of genetic determinants that govern Escherichia coli growth and persistence in lake water
Source: ISME J. 2024 Jun 14;18(1):wrae096. doi: 10.1093/ismejo/wrae096 (PMC11188689; doi:10.1093/ismejo/wrae096)
Supplement: Supplementary_Figure_S2_wrae096 [file supplementary_figure_s2_wrae096.pdf]

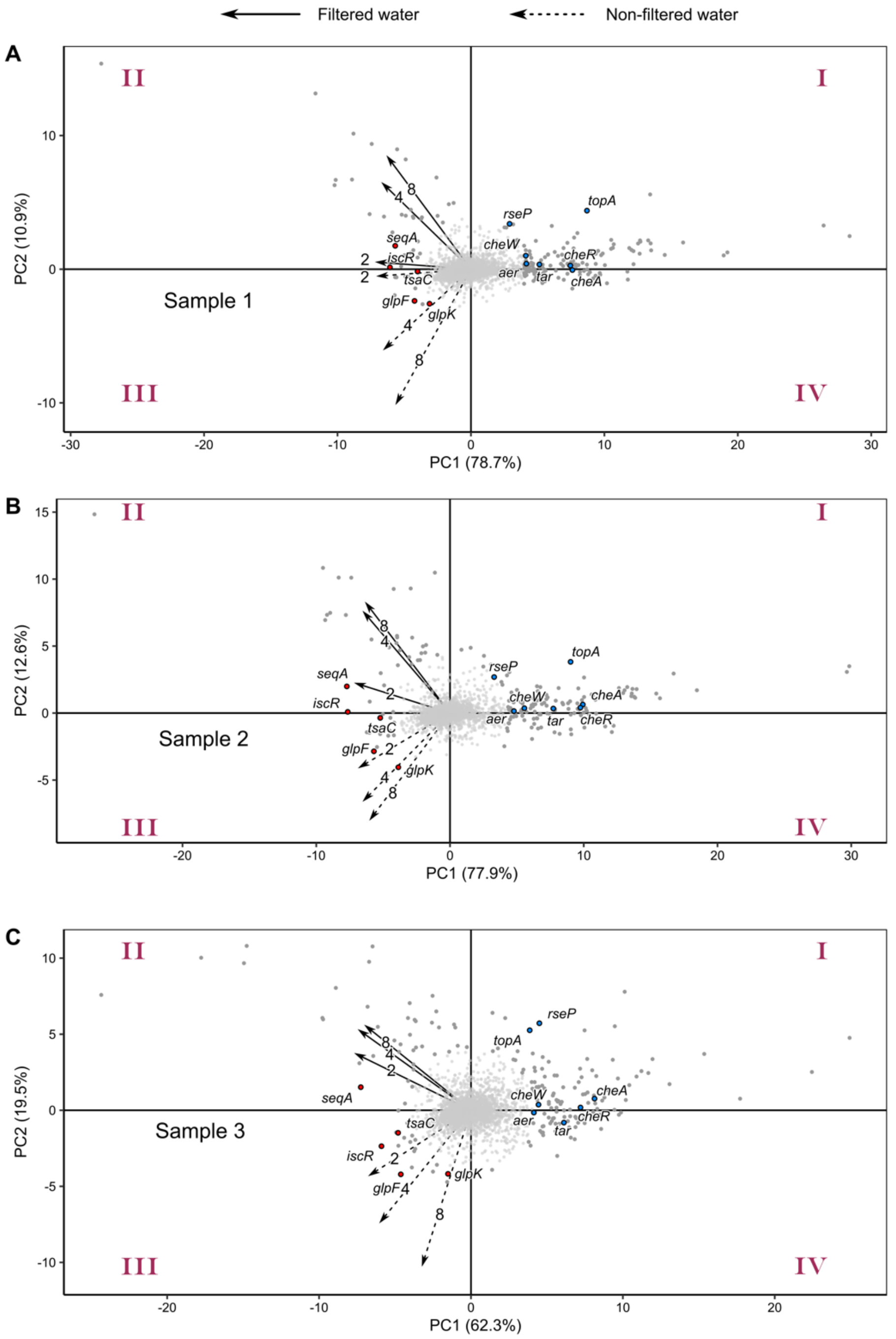

Supplementary Figure S2. Additional selected mutations affecting *E. coli* gene fitness in lake water.

Principal component analysis (PCA) of gene fitness for lake water samples #1(A), #2 (B), and #3 (C), with additional selected mutations being highlighted. Data analysis, color code and other details are as in Fig. 2.
