# Supplementary material for: Genome-wide screen of genetic determinants that govern Escherichia coli growth and persistence in lake water
Source: ISME J. 2024 Jun 14;18(1):wrae096. doi: 10.1093/ismejo/wrae096 (PMC11188689; doi:10.1093/ismejo/wrae096)
Supplement: Supplementary_Figure_S3_wrae096 [file supplementary_figure_s3_wrae096.pdf]

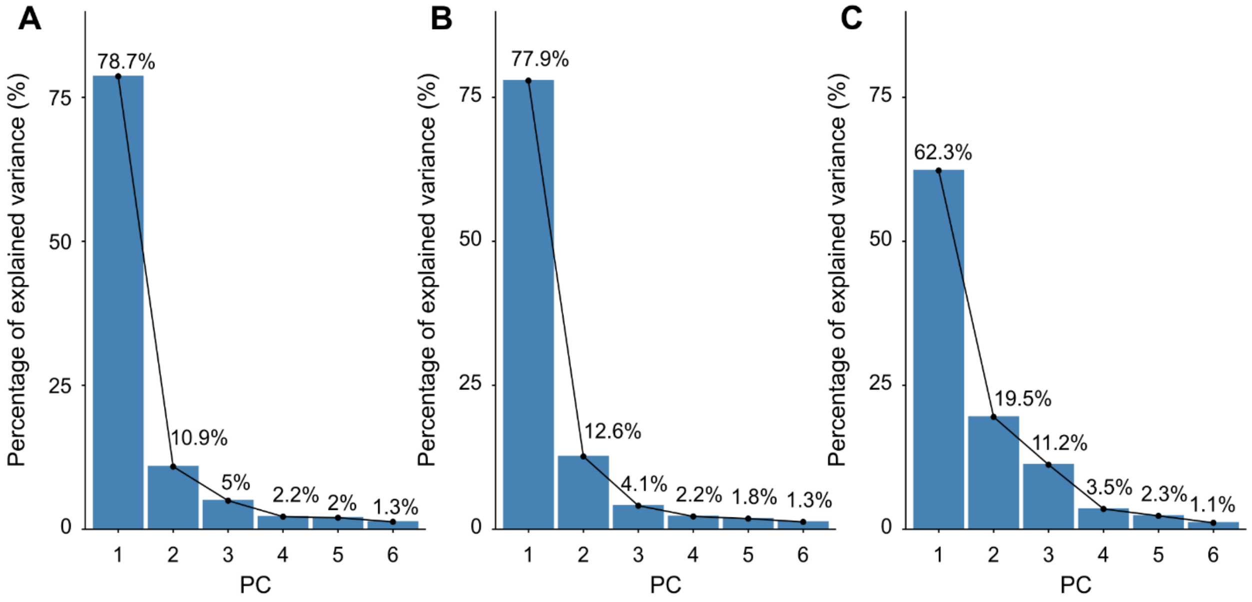

Supplementary Figure S3. Percentage of variance explained by each principal component (PC).

Results of the PCA for the water samples #1 (A), #2 (B), and #3 (C) are shown. Based on these results, the first 2 principal components were selected as they explain >80% of the variance.
