# Supplementary material for: Genome-wide screen of genetic determinants that govern Escherichia coli growth and persistence in lake water
Source: ISME J. 2024 Jun 14;18(1):wrae096. doi: 10.1093/ismejo/wrae096 (PMC11188689; doi:10.1093/ismejo/wrae096)
Supplement: Supplementary_Figure_S4_wrae096 [file supplementary_figure_s4_wrae096.pdf]

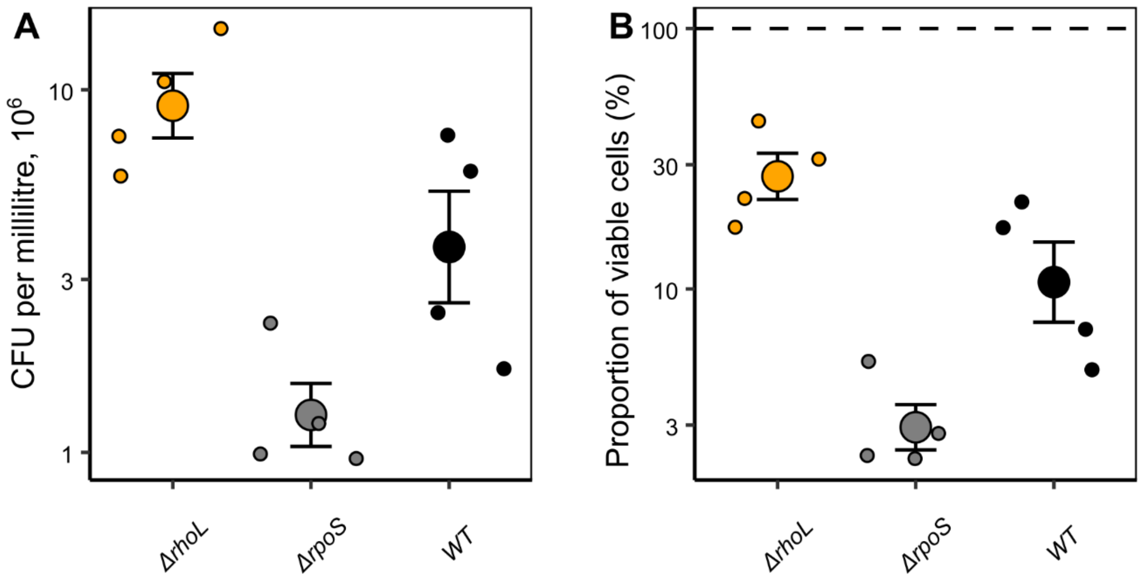

Supplementary Figure S4. Assessment of viability of the selected strains by direct colony counting.

A. Number of viable cells in the populations of selected mutants, accessed by direct colony counting, after 8 days of incubation in filtered water sample #4. Values were measured in one biological replicate, four technical replicates each.

B. Percentage of viable cells in the populations of selected mutants, accessed by direct colony counting (data from panel A) normalized by flow cytometry cell counts for the same samples. Values were measured in one biological replicate, four technical replicates each.

The small dots represent the individual values of each replicate, whereas the large dots represent the average values. Error bars show the standard error of the mean. The y-axis is logarithmic in both plots.
