# Supplementary material for: Genome-wide screen of genetic determinants that govern Escherichia coli growth and persistence in lake water
Source: ISME J. 2024 Jun 14;18(1):wrae096. doi: 10.1093/ismejo/wrae096 (PMC11188689; doi:10.1093/ismejo/wrae096)
Supplement: Supplementary_Table_S1_wrae096 [file supplementary_table_s1_wrae096.pdf]

Supplementary Table S1. Strains used in the study.

**Strain name**	**Relevant genotype**	**Reference**
**W3110 RpoS$^+$**	**Wild type**	[14]
**NT371**	**W3110 RpoS$^+$ pTrc99a-eGFP**	**This study**
**NT381**	**W3110 RpoS$^+$ Δ*rpoS***	**This study**
**NT379**	**W3110 RpoS$^+$ Δ*hdfR***	**This study**
**NT380**	**W3110 RpoS$^+$ Δ*maoP***	**This study**
**NT411**	**W3110 RpoS$^+$ Δ*rhoL***	**This study**
**NT431**	**W3110 RpoS$^+$ Δ*rbsR***	**This study**
