# Supplementary material for: Genome-wide screen of genetic determinants that govern Escherichia coli growth and persistence in lake water
Source: ISME J. 2024 Jun 14;18(1):wrae096. doi: 10.1093/ismejo/wrae096 (PMC11188689; doi:10.1093/ismejo/wrae096)
Supplement: Supplementary_Table_S2_wrae096 [file supplementary_table_s2_wrae096.pdf]

Supplementary Table S2. Parameters of taken lake water samples.

Date	Water sample	pH	Air temperature, °C	Water temperature, °C
9.05.2022	#1	7	13	12.4
19.06.2022	#2	6.5	29	18.4
6.07.2022	#3	6.5	17	16.1
1.06.2023	#4	6.5	17	14.6
8.10.2023	#5	6.5	16	13.6
